# The physiological and molecular mechanisms of N transfer in *Eucalyptus* and *Dalbergia odorifera* intercropping systems using root proteomics

**DOI:** 10.1186/s12870-021-02969-9

**Published:** 2021-04-26

**Authors:** Xianyu Yao, Liangning Liao, Yongzhen Huang, Ge Fan, Mei Yang, Shaoming Ye

**Affiliations:** 1 College of Forestry, Guangxi University, Nanning, 530004 Guangxi Province China; 2 Key Laboratory of Vegetation Restoration and Management of Degraded Ecosystems, South China Botanical Garden, Chinese Academy of Sciences, Guangzhou, 510650 Guangdong China

**Keywords:** Rhizosphere interaction, Nitrogen uptake, Nitrogen transfer, Differentially expressed proteins, Molecular mechanisms

## Abstract

**Background:**

The mixing of *Eucalyptus* with N_2_-fixing trees species (NFTs) is a frequently successful and sustainable cropping practice. In this study, we evaluated nitrogen (N) transfer and conducted a proteomic analysis of the seedlings of *Eucalyptus urophylla* × *E. grandis* (*Eucalyptus*) and an NFT, *Dalbergia* (*D.*) *odorifera,* from intercropping and monocropping systems to elucidate the physiological effects and molecular mechanisms of N transfer in mixed *Eucalyptus* and *D. odorifera* systems.

**Results:**

N transfer occurred from *D. odorifera* to *Eucalyptus* at a rate of 14.61% in the intercropping system, which increased N uptake and growth in *Eucalyptus* but inhibited growth in *D. odorifera.* There were 285 and 288 differentially expressed proteins by greater than 1.5-fold in *Eucalyptus* and *D. odorifera* roots with intercropping vs monoculture, respectively. Introduction of *D. odorifera* increased the stress resistance ability of *Eucalyptus*, while *D. odorifera* stress resistance was increased by increasing levels of jasmonic acid (JA). Additionally, the differentially expressed proteins of N metabolism, such as glutamine synthetase nodule isozyme (GS), were upregulated to enhance N competition in *Eucalyptus*. Importantly, more proteins were involved in synthetic pathways than in metabolic pathways in *Eucalyptus* because of the benefit of N transfer, and the two groups of N compound transporters were found in *Eucalyptus*; however, more functional proteins were involved in metabolic degradation in *D. odorifera*; specifically, the molecular mechanism of the transfer of N from *D. odorifera* to *Eucalyptus* was explained by proteomics.

**Conclusions:**

Our study suggests that N transfer occurred from *D. odorifera* to *Eucalyptus* and was affected by the variations in the differentially expressed proteins. We anticipate that these results can be verified in field experiments for the sustainable development of *Eucalyptus* plantations.

**Supplementary Information:**

The online version contains supplementary material available at 10.1186/s12870-021-02969-9.

## Background

Although plantations represent only 5% of the total forest area, they fulfill more than 33% of the global demand for wood products, which is anticipated to increase sharply in the coming decades [1]. *Eucalyptus* is widely planted in the tropics and subtropics and is one of the most important fast-growing trees for pulp and paper as well as the biorefinery industries [2], not only in subtropical China but also throughout the world. This tree covers 4.6 million hectares in China [3]. While *Eucalyptus* is considered to have a high commercial value, its drawbacks include high levels of nitrogen (N), phosphorus and water consumption with successive rotations, all of which decrease productivity [4]. This phenomenon occurs because N availability is often a factor limiting *Eucalyptus* growth [5], and additional N input may be required to ensure high and sustainable stand production. Therefore, fertilizers are often used in commercial eucalypt plantations, but the utilization rate of exogenous N is very low at only approximately 30% [6]. High levels of N export during harvesting every 6–7 years have led to concerns about the economic sustainability of these plantations, and current silvicultural practices result in higher N outputs than N inputs in most commercial eucalypt plantations [7], which can be expensive and potentially contribute to water eutrophication or other types of pollution [8, 9]. Thus, ecological mechanisms that occur in natural ecosystems to sustain productivity should be utilized [10], and the slight drop in productivity could be worth the reduced fertilizer costs if the difficulties of implementing mixed plantations are overcome by forest managers [1, 9].

Introducing N_2_-fixing tree species (NFTs) into *Eucalyptus* plantations may be an attractive option for sustaining high yields [11], combining ecological processes of facilitation between NFTs and non-N_2_-fixing tree species (non-NFTs) with large N inputs resulting from biological fixation of atmospheric N_2_ [8, 9]; additionally, this strategy may be a promising way to balance the soil N budget and improve soil N availability through N_2_ fixation and N recycling [12]. Many experiments have confirmed that, compared to eucalypt monocultures, mixed plantations with NFTs have the potential to increase wood production [1, 13, 14]. The positive interactions may help enhance stand productivity in mixed-species plantations with NFTs [1, 3, 15], and complementarity, which results in differences in the resource requirements between the species in the mixture when interspecific competition is lower than intraspecific competition, leads to the improved use of available resources at the stand level [16]. Nitrogen availability is likely increased for *Eucalyptus* growing in a mixture with NFTs in three ways. First, N is released by the death of plant and microbe tissues and the decomposition of NFTs and becomes available to *Eucalyptus* through the N cycles in the ecosystem [17]. Second, the soil N availability is improved to alleviate N limitations because of the N fixation by NFTs, which facilitates the growth of the target species in N-limited soils [9, 18], so more soil N may be available to *Eucalyptus* [3, 15]. Third, the mixing of *Eucalyptus* and NFTs changes the N utilization mechanism mainly through N transfer.

There has been much research on the first two ways to improve N utilization between *Eucalyptus* and NFTs [19, 20], while studies on N transfer to improve N contents have also been performed [3, 15]. To date, studies of N transfer have only concentrated on the patterns of tree growth, plant biomass, nutrient content and biological N fixation by N transfer in *Eucalyptus* and NFT plantations [1, 3, 11], and the mechanism of N transfer between *Eucalyptus* and NFTs is still poorly understand. It is thought that N derived from the atmosphere can become rapidly available to non-NFTs through root exudation or by direct transfer through common mycorrhizal networks [21], and the root system can act as a transmission tool to achieve N transfer underground. Nevertheless, studies on the molecular mechanisms of root interactions in the mixed systems of *Eucalyptus* and NFTs are lacking, and a change in plant root proteomics is crucial for the sustainable development of plant physiological metabolism in the forestry system and indirectly affects plant biological yield. Therefore, elucidating the molecular mechanisms of root development and function is important for improving plant productivity [22].

Over the past decade, management has been shown to affect subsoil root activity, and proteomics has become a common tool to resolve biotic, abiotic, physiological and biochemical processes in plants [23]. Proteomic strategies have become powerful tools that [24], when combined with complementary molecular genetics and physiological analyses, can provide a framework for understanding the molecular basis of complex biological processes [25]. However, most studies have reported that root morphological responses to a heterogeneous nutrient supply and flooding [26] or responses to environmental stresses [27, 28], such as iron sufficiency/deficiency conditions [29] and N nutrition stress [30], drought stress [31] and temperature variations [32], are the major factors affecting the physiological metabolism of the root system. Additionally, a few studies of root proteomics have been reported in intercropping systems, such as the maize/peanut [33] and bean (*Vicia faba*)/maize [34]. However, proteomic studies on N absorption in NFTs and *Eucalyptus* mixed plantations are lacking, as it is difficult to address ecological problems in a mixed system of woody plants. Thus, we hypothesized that the yield of the mixed *Eucalyptus* and NFTs was also affected by the differentially expressed proteins.

*E. urophylla × E. grandis* and an NFT, *Dalbergia odorifera*, which is grown in intercropping and monoculture systems, were used in our study. The differences between intercropping and monoculture treatments of the two species were found to result from rhizosphere effects. To elucidate the molecular basis of the *E. urophylla × E. grandis* and *D. odorifera* intercropping system, the two species were planted under the same soil conditions. Foliar ^15^N labeling was used to determine N transfer, and TMT/iTRAQ labeling was used to detect the expression levels of several N metabolism genes in the roots of the two species grown in different planting systems. The aims of the experiments were (1) to verify the competitive advantages of the two species with regard to N absorption and N transfer in the intercropping system and (2) to study the effects of differentially expressed proteins and the proteins involved in synthetic and metabolic pathways on N transfer.

## Results

### Root morphology, N uptake and N transfer

**Fig. 1:**
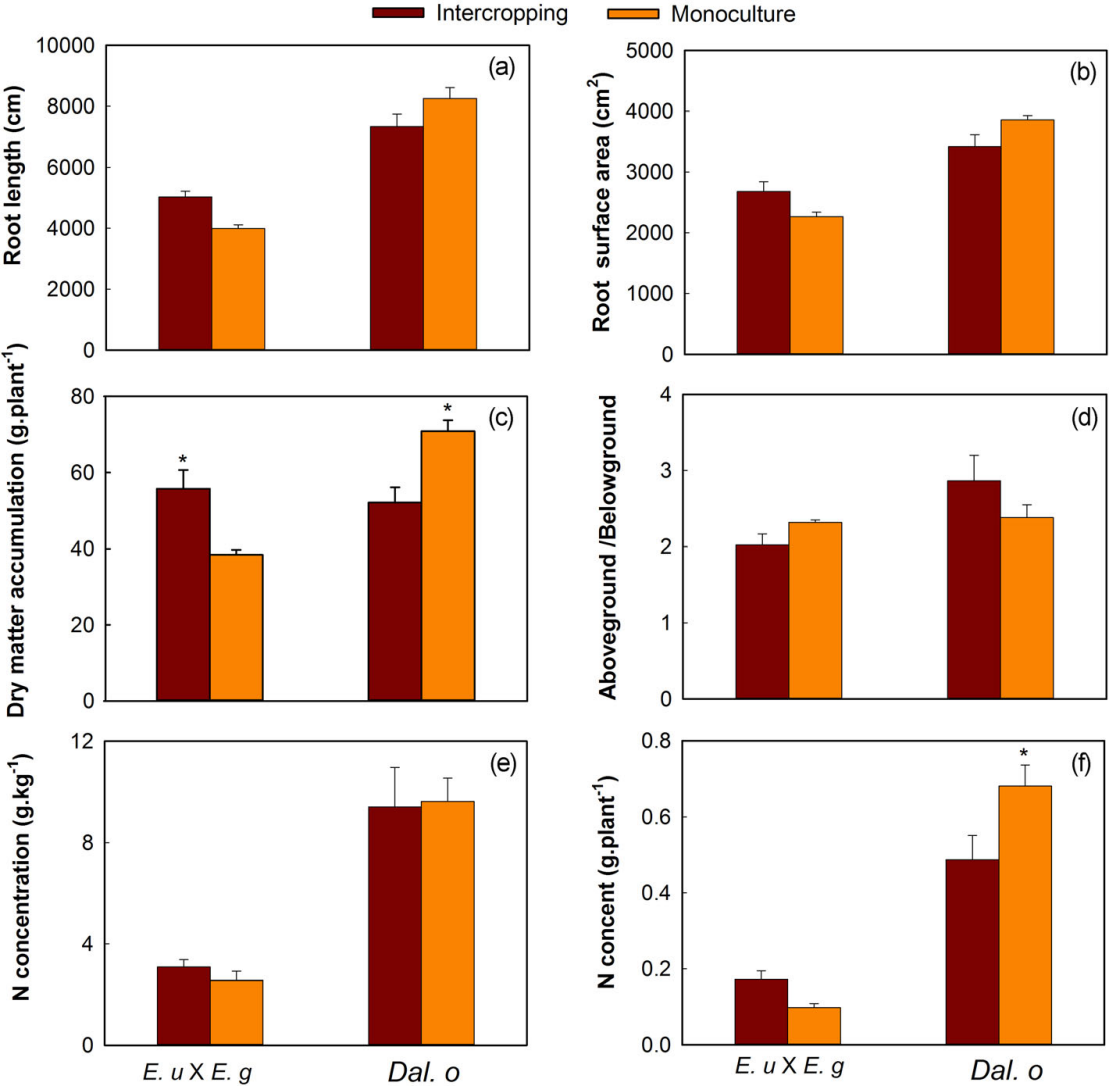
Total biomass and N content of *E. urophylla* × *E. grandis* and *D. odorifera* under different planting systems, where (**a**) is the root length, **b** is the total surface area of roots; **c** is the whole pant biomass; **d** is the ratio of aboveground to belowground on biomass; **e** is the root N concentration and (**f**) is the root N content of both tree species, respectively. Note: *E. u* × *E. g* is *E. urophylla* × *E. grandis* and *Dal. o* is *D. odorifera*

**Fig. 2:**
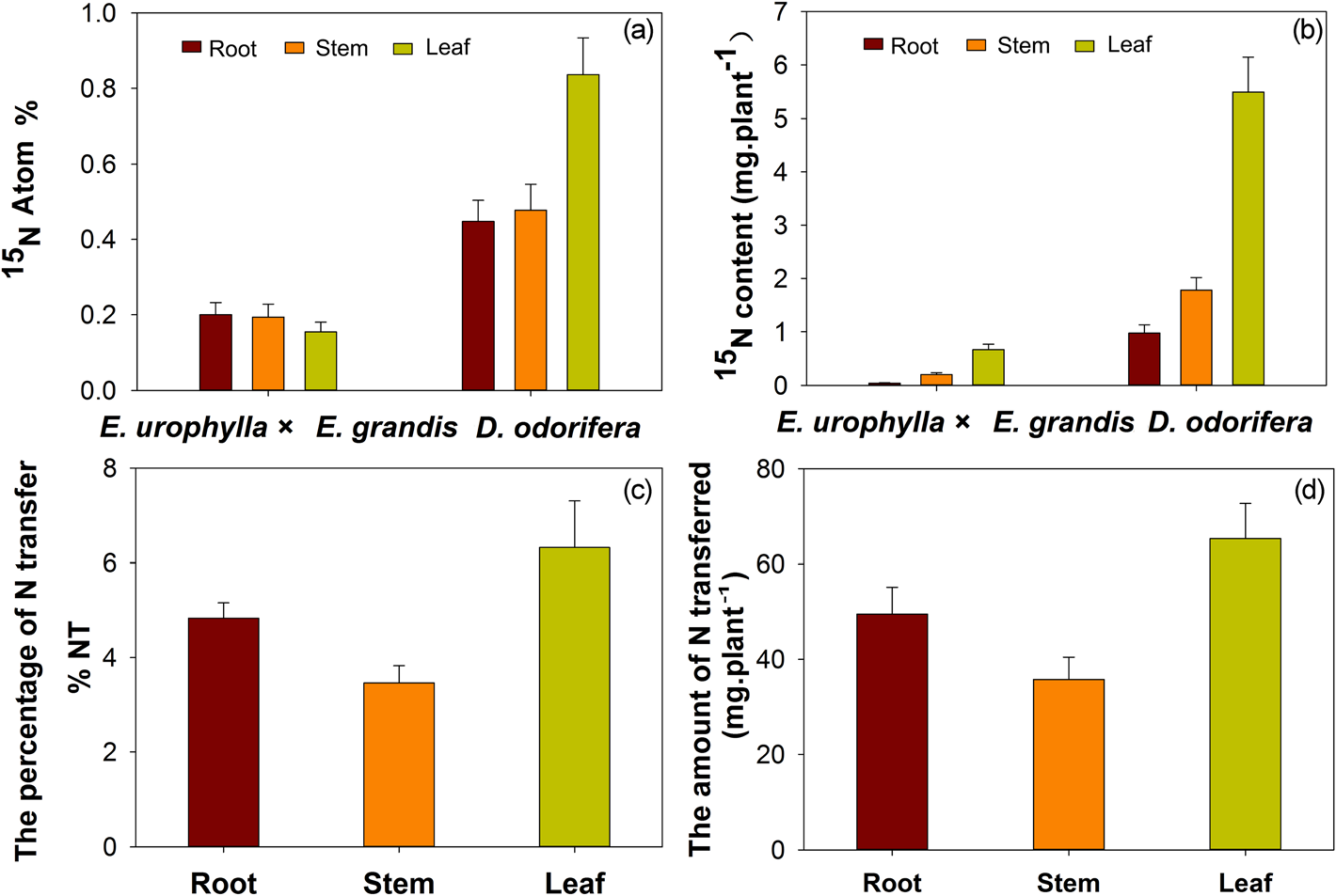
Atom % ^15^N in plant components and N transfer between *D. odorifera* and *E. urophylla × E. grandis* in an intercropping system, where (a) is ^15^N atom %, (b) is the N content in *D. odorifera *and *E. urophylla × E. grandis*, (c) is the percentage of N transfer and (d) is the amount of N transferred

The results showed that, compared to those of the monoculture, the root length, surface area, dry matter accumulation and N content of *E. urophylla* × *E. grandis* in the intercropping system significantly improved by 25.93, 18.22, 45.09 and 75.19%, respectively. Nevertheless, these parameters decreased by 11.12, 11.42, 26.43 and 28.48% in *D. odorifera*, respectively (Fig. 1a, b, c, d, e and f). In addition, ^15^N atom % in both species were detected (Fig. 2a and b), we found that N transfer occurred from *D. odorifera* to *E. urophylla* × *E. grandis* at a rate of 14.61%, which was equal to 150.62 mg of N transfer from *D. odorifera* to *E. urophylla* × *E. grandis* (Fig. 2c and d).

### Proteomic analysis revealed differentially expressed proteins

**Fig. 3:**
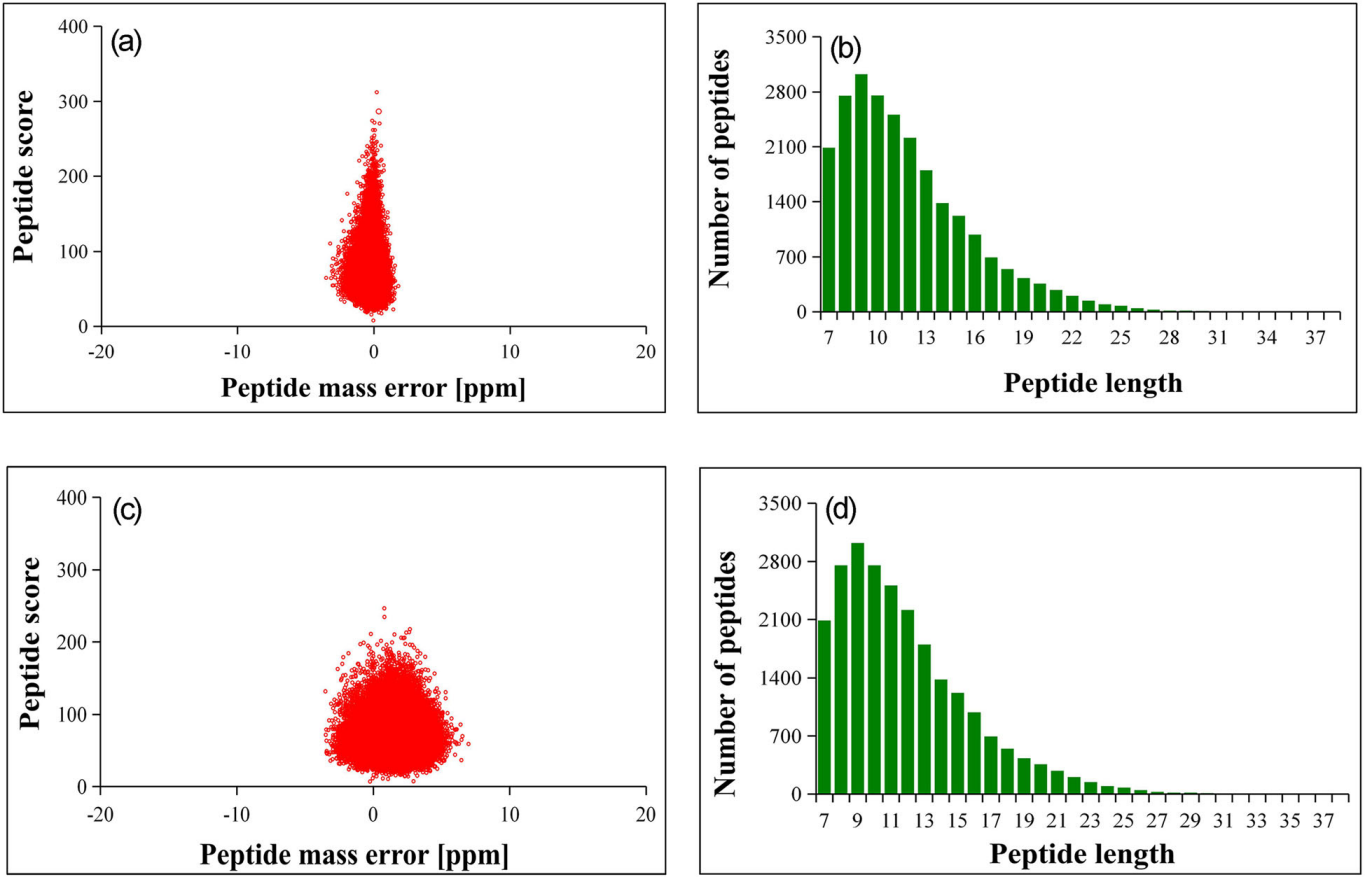
Quality control test results of mass spectrometry data, where (**a**) and (**c**) represent the peptide mass error of *E. urophylla* × *E. grandis* and *D. odorifera*, and (**b**) and (**d**) represent the peptide length of *E. urophylla* × *E. grandis* and *D. odorifera*, respectively

Our results showed that the mass error was within the requirements because the peptide mass error takes the origin as the central axis and has a range of less than 10 PPM (Fig. 3a and c). Second, the sample preparation was up to standards because most of the peptide lengths were distributed between 8 and 20 amino acid residues (Fig. 3b and d), which conforms to the law of trypsin digestion of peptides. In our study, a protein was considered differentially expressed when the protein had both a log2-fold change of more than 1.5 (upregulated) or less than 0.667 (downregulated) and a *p*-value of less than 0.05. Based on the comparison of intercropping and monoculture systems, 285 groups of differentially expressed proteins were detected in *E. urophylla × E. grandis* roots, 154 groups (54.04%) of which displayed a decreased abundance and 131 groups (45.96%) of which displayed an increased abundance. For the *D. odorifera* roots, we identified 67 groups (29.39%) of downregulated and 221 groups (70.61%) of upregulated proteins (Table 1, Tables S1 and S2).

**Table 1:** Differentially expressed protein summary of *E. urophylla* × *E. grandis*/*D. odorifera* (Filtered with the threshold values of the expression fold change and *P*-value < 0.05)

Tree species	Total spectrums	Identified proteins	Quantifiable proteins	Compare group	Regulated type	Fold change > 1.5
*E. urophylla* × *E. grandis*	254,765	5246	4414	In_E/Mo_E	downregulated	154
					upregulated	131
*D. odorifera*	224,342	5005	4136	In_D/Mo_D	downregulated	67
					upregulated	221

### Functional enrichment of the differentially quantified proteins

**Fig. 4:**
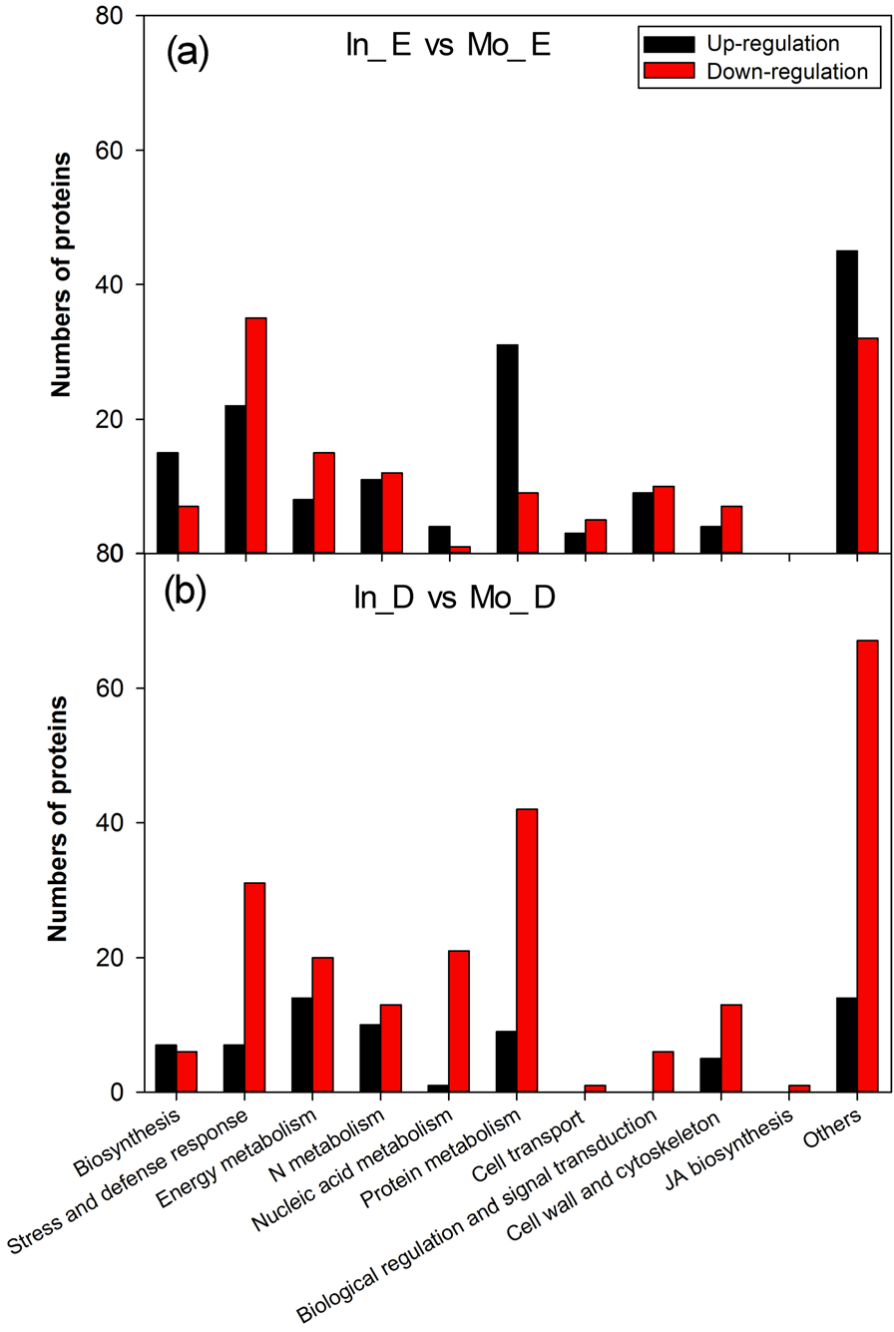
Functional category distribution of the identified proteins. **a** The proteins of *E. urophylla* × *E. grandis* in intercropping vs monoculture, and **b** *D. odorifera* in intercropping vs monoculture. In_ E and Mo_E represent intercropped and monoculture *E. urophylla × E. grandis*, and In_D and Mo_D represent intercropped and monoculture *D. odorifera*, respectively

In our study, the results showed that the differentially expressed proteins with a fold change of at least 1.5 were related to biosynthesis, stress and defense responses, carbohydrate and energy metabolism, nucleic acid metabolism, protein metabolism cell transport, biological regulation and signal transduction, cell wall and cytoskeleton metabolism, jasmonic acid (JA) biosynthesis and others. For intercropping vs monoculture of *Eucalyptus*, the proteins of biosynthesis, nucleic acid metabolism and cell transport were upregulated and the others downregulated (Fig. 4a). For *D. odorifera*, most of the proteins were downregulated except those with functions in biosynthesis for intercropping vs. monoculture (Fig. 4b).

**Fig. 5:**
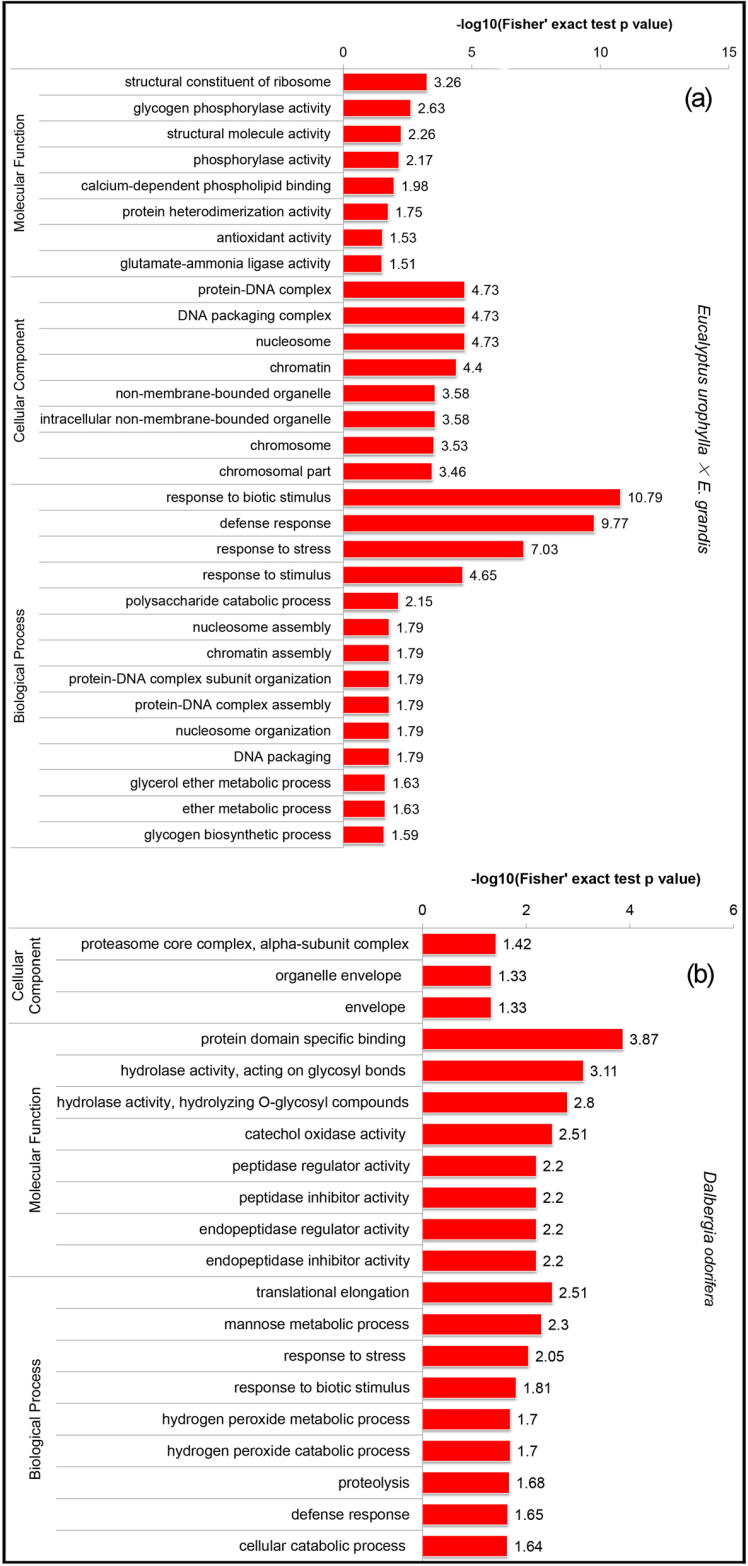
GO-based enrichment analysis of all the proteins from *E. urophylla* × *E. grandis* and *D. odorifera.* **a** *E. urophylla* × *E. grandis* intercropping vs. monoculture, and **b** *D. odorifera* intercropping vs monoculture*.* In_ E and Mo_E represent intercropped and monoculture *E. urophylla × E. grandis*, and In_D and Mo_D represent intercropped and monoculture *D. odorifera*, respectively

After the proteins were assigned to different categories, their quantities were calculated via the -log10 (*p-value*) method. For *E. urophylla × E. grandis* intercropping vs monoculture, the difference in protein content varied from 1.51 for those related to glutamate-ammonia ligase activity to 10.79 for response to a biotic stimulus (Fig. 5a). Nevertheless, for *D. odorifera*, the protein content varied from 1.33 for envelope to 3.87 for domain-specific binding proteins (Fig. 5b).

### KEGG pathway analysis of the differentially expressed proteins

**Fig. 6:**
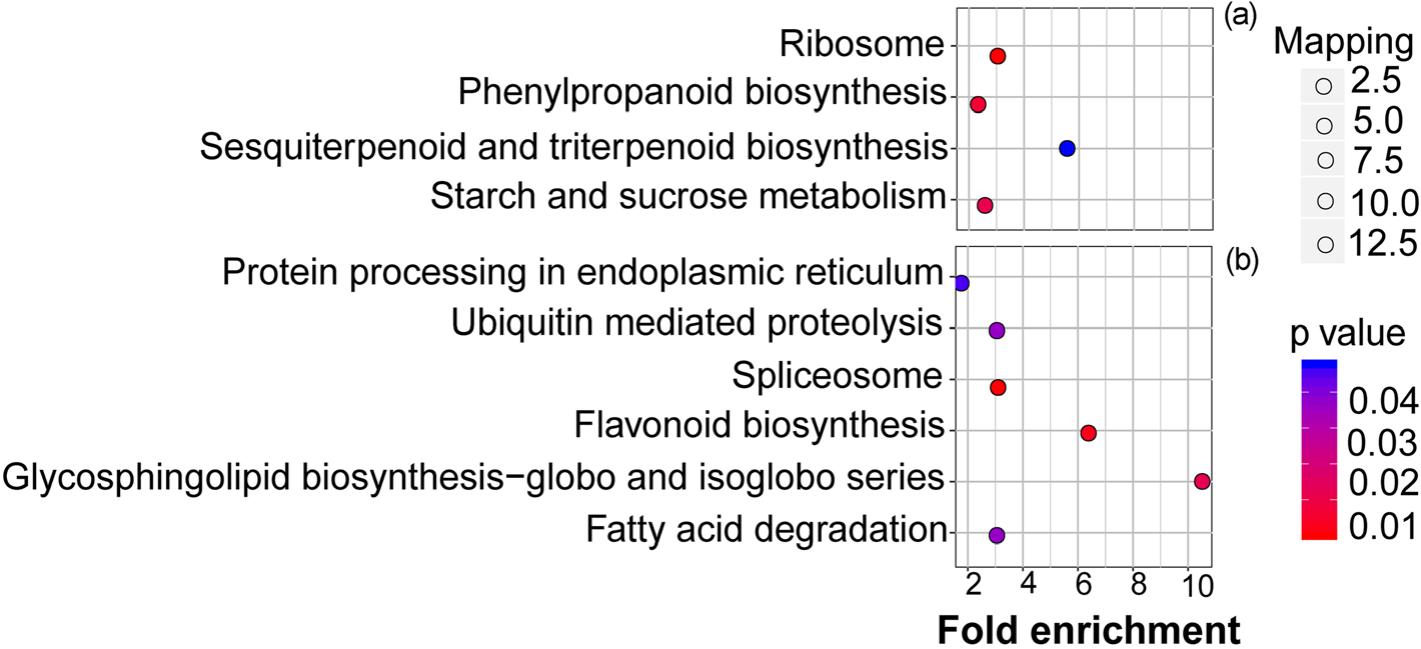
Differential proteins involved in KEGG pathways. **a** *E. grandis* intercropping vs monoculture and **b** *D. odorifera* intercropping vs monoculture

Four pathways were identified for *E. urophylla × E. grandis* proteins, e.g., ribosome (13 groups of proteins, *p* < 0.05), phenylpropanoid biosynthesis (9 groups of proteins), starch and sucrose metabolism (6 groups of proteins) and sesquiterpenoid and triterpenoid biosynthesis (2 groups of proteins) (Fig. 6a and Table S3). For *D. odorifera* proteins, six pathways were identified, which included spliceosome (10 groups of proteins), flavonoid biosynthesis (4 groups of proteins), glycosphingolipid biosynthesis-globo and isoglobo series (2 groups of proteins), ubiquitin-mediated proteolysis (4 groups of proteins), fatty acid degradation (4 groups of proteins), and protein processing in the endoplasmic reticulum (8 groups of proteins) (Fig. 6b and Table S4).

**Fig. 7:**
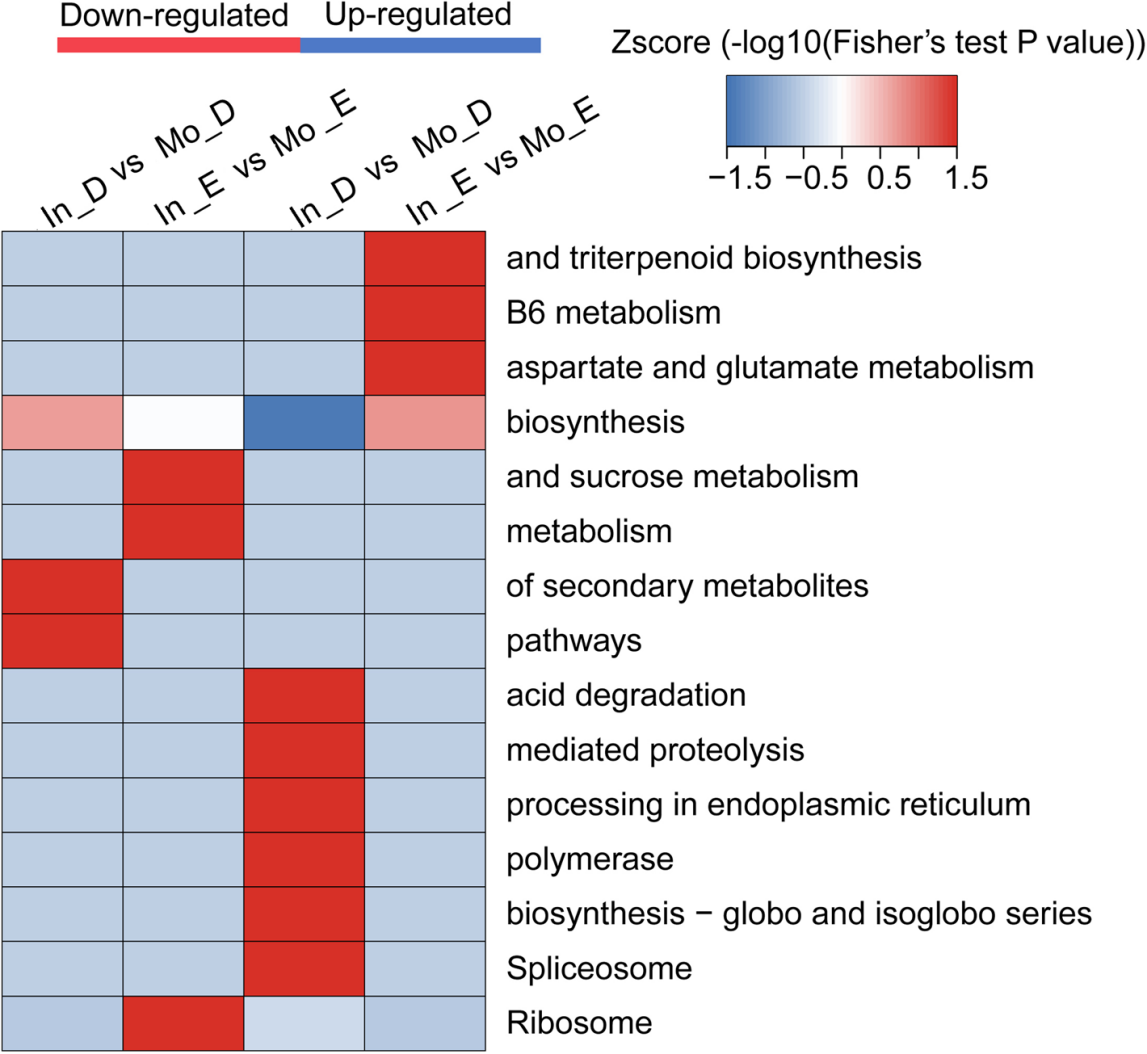
KEGG pathway enrichment-based clustering analysis of all the identified proteins. In_ E and Mo_E represent intercropped and monoculture *E. urophylla × E. grandis*, and In_D and Mo_D represent intercropped and monoculture *D. odorifera*, respectively

The quantities were calculated via the -log10 (*p*-value) method, similar to the functional enrichment. For intercropping vs. monoculture of *E. urophylla* × *E. grandis*, the abundance of proteins related to sucrose metabolism, metabolism, ribosome, triterpenoid biosynthesis, B6 metabolism, and aspartate and glutamate metabolism was significantly increased. Nevertheless, *D. odorifera* showed different results, as acid degradation, mediated proteolysis, processing in the endoplasmic reticulum, polymerase, biosynthesis-globo and isoglobo series, spliceosome, and secondary metabolism pathways were significantly enriched (Fig. 7).

### Differentially expressed proteins by PRM

The differentially expressed proteins identified by iTRAQ were validated by PRM. For analysis by PRM, we selected 4 groups of proteins involved in N metabolism and physiological metabolism, which were common to both species. As shown in Table 2, the gene expression levels of the four groups for both tree species, were well matched. The mean expression levels of the peroxidase and N metabolism were all higher in the intercropping *Eucalyptus* than in the monoculture, and all of these proteins demonstrated a higher level in intercropping *D. odorifera.* The t-test revealed some differences in the levels of the four target proteins under these two different conditions, which was exactly consistent with the trend observed when the protein levels were quantified by iTRAQ.

**Table 2:** Results of relatively quantitative analysis of target peptide by PRM

Species	Protein name		Mo_average	In_average	Ratio_In/Mo	*P*-value (T-test)
*E. urophylla* × *E. grandis*	Peroxidase	A0A059AL91	0.619	1.381	2.231	0.00566
	Ribosomal protein	A0A059BJR3	1.365	0.635	0.466	0.00012
	N metabolize	A0A059BMH2	0.646	1.354	2.097	0.00256
	Transport protein	A0A059DEJ5	1.265	0.735	0.581	0.00063
*D. odorifera*	Peroxidase	TRINITY_DN34877	0.671	1.329	1.979	0.00001
	Ribosomal protein	TRINITY_DN31489	0.764	1.236	1.619	0.00212
	N metabolize	TRINITY_DN39873	0.467	1.533	3.279	0.00000
	Transport protein	TRINITY_DN49158	0.626	1.374	2.197	0.00426

To examine the correlation and accuracy between the data from the iTRAQ and PRM analyses, we compared the correlation between the protein expression levels obtained by iTRAQ with that obtained by PRM. The results show that, for the iTRAQ and PRM data, the peroxidase and ribosomal protein levels were significantly correlated (R^2^ > 0.75), whereas the N metabolism and transport protein levels were not significantly correlated (0.50 ≤ R^2^ < 0.75).

## Discussion

Improved nutrient utilization is one of the major advantages of legume/non-legume intercropping systems [35], and it depends mainly on the ability of the roots to acquire external resources for plant survival in different environments and to adapt to external disturbances [36]. In this study, our results showed that interspecific rhizosphere effects significantly improved N uptake and promoted the development of *E. urophylla* × *E. grandis*, but the effect on *D. odorifera* in the intercropping systems was limited, possibly because the root exudates by *Eucalyptus* had an allelopathic effect on *D. odorifera* [37]. In addition, the transferred N provided key N resource for *Eucalyptus* and significantly improved seedling physiological performance by increasing plant growth and nutrient storage reserves for subsequent root growth [38]. Nevertheless, the limitations on the root growth of *D. odorifera* may have also been caused by N transfer and reduced its own N nutrients [3, 15]. Our results also emphasized that planting NFTs might be an attractive option for maintaining the N fertility of soils planted with *Eucalyptus* [4, 39]. In addition to the soil and root N concentration [3], N transfer was most likely caused by differentially expressed proteins of plant roots.

In our study, there were 285 and 288 differentially expressed proteins of greater than 1.5-fold detected in *E. urophylla* × *E. grandis* and *D. odorifera* roots in the intercropping and monoculture systems, respectively (Table 1). From the differentially expressed proteins, the identified proteins were further categorized on the basis of their putative functions. The proteins with a higher abundance in intercropping were mainly involved in stress tolerance (26.7%) and metabolism proteins (16.0%) of *Eucalyptus* (Fig. 4a), while most of differentially expressed proteins that were upregulated (76.7%) in intercropped *D. odorifera* (Table 1), especially metabolic proteins, involved upregulated proteins at a rate of 43.4% (Fig. 4b). In addition, among the 4 group proteins in common for both trees analyzed by PMR, the ratio of the downregulated root protein groups decreased, but that of the upregulated protein groups increased (Table 2). The difference between gene expression level and protein abundance was caused by post-translational modifications. The use of proteomics to identify key proteins associated with metabolism (e.g., N metabolism and amino acid metabolism) and synthesis processes can provide insight into the mechanisms of N transfer.

### The positive effects of *Eucalyptus* likely arise from differentially expressed proteins in the intercropping system

Comparative proteomics of roots are frequently used to investigate growth, especially physiological stress response mechanisms in plants [40]. Previous studies have shown that monoculture *Eucalyptus* may be less effective at decreasing diseases and enhancing disease suppression than intercropped *Eucalyptus* [35, 41]. Our proteomic study indicated that some crucial proteins of stress- and defense-related proteins increased in monoculture *E. urophylla* × *E. grandis* roots in response to oxidative stress, including those related to response to biotic stimulus, defense response, response to stress, and response to stimulus, among others (Figs. 4 and 5). For example, higher levels of peroxidase in intercropped *E. urophylla* × *E. grandis* were detected (A0A059AL91, Table 2), which not only prevented active injury but also degraded auxin (probable indole-3-acetic acid-amido synthetase GH3.1 IAA) and reactive oxygen species (ROS) [34]. These changes further promoted the rebalancing of the hormonal system and regulated the formation of adventitious roots and lateral roots to adapt to environmental stresses [42]. However, peroxidase 4-like (Additional file 2: TRINITY_DN34591, Table S2-No. 55) and nodulin-13-like isoform X1 (Additional file 2: TRINITY_DN40711, Table S2-No. 112) were downregulated in intercropped *D. odorifera*, which may also indicate inhibitory effects on *D. odorifera* in the intercropping system. Additionally, alcohol dehydrogenase was upregulated in monoculture *E. urophylla* × *E. grandis* when plants were under stress (Additional file 1: A0A058ZYN2, Table S1-No. 160), and then, the nucleobase-ascorbate transporter showed higher expression to regulate the H_2_O_2_ content in plants to improve their stress resistance [43]. *Eucalyptus* growth would benefit from the changes in these proteins because of the introduction of NFTs.

In *E. urophylla* × *E. grandis* roots, glutathione S-transferase (GST) (Additional file 1: A0A059AX10, Table S1- No. 49) and the homolog glutathione S-transferase U25 (Additional file 1: A0A058ZUA9, Table S1-No. 60) increased in the intercropping system compared to the monoculture system; these compounds play a crucial role in cell detoxification and stress tolerance in plants [33], increasing toxin removal through increased enzyme levels [44]. In addition, high contents of gibberellins (Additional file 1: A0A059A3Y5, Table S1-No. 6) and thioredoxin (Additional file 1: A0A059BPT5, Table S1-No. 99) were found in intercropping *E. urophylla* × *E. grandis* roots, which can ameliorate plant diseases. Therefore, intercropping vs monoculture revealed an advantage of *Eucalyptus* growth and provided *Eucalyptus* with stronger stress resistance [33, 34] through protein regulation. Nevertheless, for intercropping vs monoculture *D. odorifera*, the gibberellins (Additional file 2: TRINITY_DN35143, Table S2-No. 61) were downregulated because of the increasing stress resistance. Under such circumstances, jasmonic acid (JA) signaling, which plays an important role in the self-protective responses against opportunistic damage, was upregulated [33]. Overall, we suggest that the advantages of *Eucalyptus* interactions in intercropping systems may improve its ecological adaptation compared with monoculture systems, while the advantages of *Eucalyptus* in these interactions likely represents a key signal for N transfer from *D. odorifera.*

### Regulation of N transfer between *Eucalyptus* and *D. odorifera* from a proteomics perspective

As shown in previous studies, legume-derived N is transferred to neighboring *Eucalyptus* plants [3, 15]. In our study, we demonstrated that N transfer occurred from *D. odorifera* to *E. urophylla* × *E. grandis* at a rate of 14.61%, which was equal to an enhancement of 150.62 mg N in *E. urophylla* × *E. grandis* (Fig. 2) and was related to differential protein expression. Proteins related to N compound transport, which promote the synthesis and transport of N in plants, were found at a higher abundance in intercropped *E. urophylla* × *E. grandis*. We believe that the higher abundance of N transport proteins is beneficial to the synthesis and absorption of N by *E. urophylla* × *E. grandis*. Importantly, through KEGG pathway analysis of intercropping vs monoculture, more proteins with functions in synthesis (11 groups) than metabolic functions (6 groups) were found in *E. urophylla* × *E. grandis*, while the opposite result was found in *D. odorifera* (20 groups for metabolic and 6 groups for synthesis functions) (Figs. 6 and 7, Tables S3 and S4); these may represent a key signal for N transfer from *D. odorifera* to *Eucalyptus*. N transfer to the associated plants from NFTs occurred through the direct excretion of N compounds from active nodulated roots [44, 45] or the exudation of soluble N compounds from the decomposition of dead plant parts [46], resulting in more metabolic pathways in *D. odorifera* with intercropping vs monoculture. Therefore, to meet the conditions for N transfer in the intercropping system, more N compounds were secreted by intercropped *D. odorifera* with a greater number of metabolic proteins, while synthesis probably occurred in intercropped *Eucalyptus* after absorption of the N compounds from *D. odorifera*.

In addition to these intuitive representations, N transfer was related to the proteomics of N metabolism and N assimilation proteins in both species in our study. The results showed that the abundance of N-metabolized proteins changed in the two species via root interactions, with high protein levels of glutamate dehydrogenase (GDH) and glutamine synthetase nodule isozyme (GS) (Additional file 1: A0A059BTT8, Table S1-No. 118) in *E. urophylla* × *E. grandis* with intercropping vs monoculture (upregulated by 1.5-fold). When a plant absorbs inorganic N from the soil, GS and GDH are used to first converted it into organic nitrogen. Thus, GS can channel all of the N in the plant through the catalysis of reactions, and it is a key enzyme in the N assimilation and metabolism pathways [47, 48]. A previous study showed that GS in plants improved plant growth and productivity [33] due to increases in the abundance of N metabolism proteins, such as GDH and GS, by the rhizosphere effect [34]. Our results also showed that rhizosphere effects promoted N assimilation and productivity in *E. urophylla* × *E. grandis* roots, but restricted those in *D. odorifera*. More importantly, sucrose synthase (SuSy) was found in *D. odorifera* roots (Additional file 2: TRINITY_DN40013, Table S2-No. 106) and was downregulated in the intercropping system. Gordon et al. (1999) suggested that sucrose metabolism regulates and controls SuSy expression in NFTs to alter the N fixation efficiency [49]. Our previous research has confirmed that there was a positive correlation between N transfer and N_2_-fixation in NFTs [3, 50]; thus, the change in SuSy suggested that N transfer occurred between *D. odorifera* and *E. urophylla* × *E. grandis*. All these results indicated that GS, GDH and SuSy are the key signals for N transfer in the intercropping system.

Amino acid metabolism is also a key factor reflecting N metabolism and transport between *E. urophylla* × *E. grandis* and *D. odorifera*. N-deficient root tissues are capable of rapidly promoting the decomposition of amino acids and the synthesis of new amino acids through aminotransferase, which mediates the level of N metabolism [51]. Peptide-N4-(N-acetyl-beta-glucosaminyl) asparagine amidase A (Additional file 1: A0A059A9F1, Table S1-No. 205) and aspartyl protease AED3 isoform X2 (Additional file 1: A0A059AD87, Table S1-No. 195) showed a lower accumulation in the intercropping system compared to the monoculture, which indicated N transfer to *Eucalyptus* and alleviated the N deficiency in the intercropping system. Isocitrate dehydrogenase (ITD) induces isocitrate oxidative decarboxylation to produce A-ketoglutaric acid, and NADP^+^-ITD in the cytoplasm is connected to the GS/GAGOT cycle, providing a carbon skeleton and NADPH for ammonium assimilation. Here, we found that the NADP-dependent malic enzyme isoform X1 in was higher *E. urophylla* × *E. grandis* roots under intercropping than monoculture, but the change was less than 1.5-fold, indicating that the direction of carbon skeleton flow to N assimilation was enhanced. Additionally, we also found higher expression levels of transaminase, which catalyzes the amino transfer between amino acids and ketoacids, in the two species in the intercropping system vs. monoculture. For example, alanine-glyoxylate aminotransferase (Additional file 1: A0A059A1E9, Table S1-No. 108) and D-amino-acid transaminase (A0A059BMH2, Table 2 and Additional file 1: Table S1-No. 131) of *E. urophylla* × *E. grandis* was upregulated; for *D. odorifera*, putative branched-chain-amino-acid aminotransferase 7 isoform X1 (TRINITY_DN45090), acetylornithine aminotransferase (Additional file 2: TRINITY_DN44984, Table S2-No. 163) and tryptophan aminotransferase-related protein 4-like (TRINITY_DN39873, Table 2 and Additional file 2: Table S2-No. 105) were upregulated. These results emphasized that N assimilation was enhanced in plant roots, especially those of *Eucalyptus*, in the intercropping system, which explains the indirect indication of N transfer between the two species.

### Potential effects of different glycolytic pathways and the TCA cycle on N transfer

Previous studies have suggested that N shortage also causes abundant changes in proteins involved in glycolysis and the TCA cycle [51]. The glycolytic pathway degrades sugars to pyruvate [52], and when the mitochondrial pyruvate carrier is reduced, the pyruvate carrier protein on the mitochondrial intima is decreased. Pyruvate kinase, the rate-limiting enzyme for transferring the high-energy phosphate from phosphoenolpyruvate to ADP and producing ATP, was found at a higher level in *D. odorifera* roots with intercropping vs monoculture, possibly due to the N in the intercropped *D. odorifera* transfer to *E. urophylla* × *E. grandis*. In addition, N-deficient tissues were capable of rapidly incorporating acetate into certain fatty acids, particularly palmitic and oleic acids. The degradation of those compounds to yield acetyl-CoA (AC) is termed ketogenic because these substances can be used to synthesize fatty acids or ketone bodies [51]. The enhancement of glycolysis will lead to the accumulation of AC during the TCA cycle, resulting in large amounts of ATP in response to the acute N deficiency [53]. In our study, AC (Additional file 2: TRINITY_DN48754, Table S2-No. 238) was found at a higher level in intercropped than in monoculture *D. odorifera* in response N deficiency after N transfer. Moreover, the TCA cycle, the key process in the energy cycle and the ultimate metabolic pathway for nutrients, includes three key enzymes: citrate synthase (CS), ITD and ketoglutarate dehydrogenase alpha (KLDA) [33]. CS (A0A059AKY9, A0A059B6K2, A0A059BEH3, A0A059D8E5) was found only in the *E. urophylla* × *E. grandis* roots, but it was changed less than 1.5-fold with intercropping vs monoculture. ATP-citrate synthase alpha chain protein 1 (A0A059D8E5) was increased in intercropped *E. urophylla* × *E. grandis*, which facilitates the N transfer from *D. odorifera* to *Eucalyptus* to promote chlorophyll synthesis. NADP-dependent malic enzymes catalyze the oxidative decarboxylation of malic acid pyruvate, participate in the glycolytic pathway and TCA cycle [54] and were upregulated in intercropped *E. urophylla* × *E. grandis* (Additional file 1: A0A059CL16, Table S1-No. 80; A0A059CS67, Table S1-No. 72), resulting in changes in N metabolism through a coordinate regulation of the C and N metabolic pathways [55]. This phenomenon was largely due to the increase in N content by the N transfer from *D. odorifera* to *E. urophylla* × *E. grandis*.

In the TCA cycle, the key enzyme that converts citrate into isocitrate is aconitate hydratase, and in the glycolytic and gluconeogenesis pathways, enolase is the key enzyme responsible for catalyzing the reversible dehydration of 2-phospho-D-glycerate into phosphoenolpyruvate [33]; however, there were no significant differences in these two enzymes in either species between the intercroppin*g* and monoculture treatments. However, ribosomes are varied structurally distinct proteins and play a significant role in translational regulation and N metabolism [56], which was upregulated in intercropped *D. odorifera* (TRINITY_DN31489, Table 2) but downregulated in intercropped *Eucalyptus* (A0A059BJR3, Table 2) by iTRAQ and by PRM. This result is consistent with the findings for the KDGG pathways, i.e., the stronger metabolic function in intercropping *D. odorifera* prompted N transfer to *Eucalyptus*.

## Conclusion

The present results encourage us to recommend *E. urophylla* × *E. grandis*/*D. odorifera* plantations. N transfer occurred from *D. odorifera* to *E. urophylla* × *E. grandis* and established a beneficial cycle between nutrient provision and *E. urophylla* × *E. grandis* growth to provide biomass, but N uptake was not changed by the rhizosphere effects in *D. odorifera*. Rhizosphere effects promoted N assimilation and N transfer by enhancing the levels of some protein species, such as ATP synthase, GS and GDH. Notably, *E. urophylla* × *E. grandis* was beneficial in the process of N transfer, and there were more differentially expressed proteins involved in the synthesis pathways than in metabolism pathways, but the opposite result was observed for *D. odorifera.* The two groups of N compound transporters were found in *E. urophylla* × *E. grandis* to improve N assimilation and synthesis; i.e., the molecular mechanism of the N transfer from *D. odorifera* to *E. urophylla* × *E. grandis* was explained by proteomics in our study. However, studies on the possible benefits of N transfer in this system should be provided to evaluate the long-term influence on productivity. Therefore, more trials focused on these environmental conditions, analytical methods and field experimentation assessments are needed to verify these findings and recommendations.

## Methods

### Experimental site and design

#### Experiment 1

The experiments were carried out in the greenhouse at Guangxi University, China (108°17′30.3″E, 22°51′4.79″N) on May 18, 2017, with air temperatures ranging from 21 °C to 28 °C. One *D. odorifera* plant was intercropped with one *E. urophylla × E. grandis* plant in each pot (50 cm diameter and 45 cm depth), and *D. odorifera* and *E. urophylla* × *E. grandis* monocultures represented the controls in our trial, i.e., two *D. odorifera* or two *E. urophylla* × *E. grandis* were planted in each pot. All the plant materials are very common in south China, and we complied with institutional, national or international guidelines in our study. The plant materials were obtained with permission from the commercial nursery of Ba Gui, Naning. The soil, previously planted with *Pinus massoniana* was collected at Liang Fengjiang Experimental Station, Nanning, China. The characteristics were as follows: 1.22 g total N kg^− 1^, 0.57 g total P kg^− 1^, 11.85 g K kg^− 1^, and pH 4.65. The soil was dried and mixed with perlite at a soil: perlite ratio of 25:1 to maintain water permeability in our study.

To avoid nutrient loss, plastic leakproof trays were placed at the bottom of the pot. The plants were watered to maintain the soil moisture at 40–80% of the water holding capacity during the entire growth stage. All treatments were applied in a complete random design with three replicates for each treatment.

### Experiment 2 (^15^N labeling)

In this experiment, the planting conditions were exactly the same as in *experiment 1*. We used PVC cylinders (80 by 120 cm) open at both ends to enclose the *D. odorifera* canopy, the leaves of which were sprayed with ^15^N-labeled urea as described by Yao et al. [3]. A 0.75% (m/m) solution of ^15^N-labeled urea with 10.32 atom % ^15^N was used to label the surface of the *D. odorifera* leaves, and thereafter, the leaves were immediately covered with sealable polythene bags until the next day to avoid ^15^N contamination of the associated *E. urophylla* × *E. grandis* or soil. The soil surface was covered by two layers of plastic film with a sponge above them to prevent ^15^N contamination from runoff of the ^15^N-labeled solution during foliar feeding. All ^15^N-labeling processes were strictly controlled to ensure that there was no ^15^N contamination of the soil or the *E. urophylla* × *E. grandis* leaves.

### Root determination and N analysis

After 6 months, 1 g of the root tips of *E. urophylla × E. grandis* and *D. odorifera* was collected in December 2017, and each root was washed with deionized water and stored in liquid N (at − 80 °C) for 10 min for further analyses. At harvest, plants from the intercropping were separated into *E. urophylla × E. grandis* and *D. odorifera*. The roots of the two species were separated by hand and washed carefully to remove the soil. Root length and surface area were scanned by an Epson root scanner and were used to obtain image analysis by WinRHIZON Pro. Then, the harvested material was dried at 60 °C until a constant dry weight was obtained. The dried root material was ground in a ball mill and passed through a 0.2-mm screen, and the total N content was determined by using a continuous flow chemical analyzer (AA3, SEAL Analytical, Norderstedt, Germany).

### Protein analysis

#### Protein extraction and preparation

The samples from experiment 1 were taken from a − 80 °C freezer, the appropriate amount of tissue sample was added to a liquid N-precooled mortar, and the liquid N sample was fully ground to a powder. The soluble protein was extracted following the procedure developed by Neilson et al. and Guo et al. [57, 58]: first, we added four volumes of lysis buffer (10 mM dithiothreitol (DTT), 1% Protease Inhibitor Cocktail and 2 mM EDTA) to 400 mg of lyophilized root powder, and then vortex centrifugation was performed at 20,000 g and 4 °C for 10 min. Finally, the protein was precipitated with cold 20% TCA at 4 °C for 2 h, and the supernatant was discarded after centrifugation at 12,000 g and 4 °C for 10 min. The remaining precipitate was washed with cold acetone three times, and 8 M urea was added to redissolve the protein; then, a BCA kit was used according to the manufacturer’s instructions to determine the protein concentration.

#### TMT/iTRAQ labeling

The protein solution was reduced with 5 mM dithiothreitol at 56 °C for 30 min and alkylated with 11 mM iodoacetamide in the dark and at room temperature for 15 min. The protein sample was diluted by adding 100 mM TEAB to urea at a concentration of less than 2 M. After this, for the first digestion, trypsin was added at a 1:50 trypsin-to-protein mass ratio overnight, and a second digestion was performed at a 1:100 mass ratio of trypsin-to-protein for 4 h. The peptides were desalted and vacuum-dried after trypsin digestion. Finally, we reconstituted the sample in 0.5 M TEAB according to the manufacturer’s protocol and processed with the TMT kit/iTRAQ kit [59, 60].

#### LC-MS/MS analysis

The tryptic peptides were dissolved in 0.1% formic acid (solvent A) and separated by the EASY-NLC 1000 UPLL system. Liquid A was an aqueous solution containing 0.1% formic acid and 2% acetonitrile. The liquid gradient settings were as follows: 6–24% solvent B for 0–26 min; 24–33% for 26–34 min; 33–75% for 34–37 min; 75% for 37–40 min; the flow rate was maintained at a constant flow rate of 700 NL/min.

The peptides were subjected to an NSI source followed by tandem mass spectrometry (MS/MS) in a Q Exactive™ Plus (Thermo) coupled to the ultra-performance liquid chromatograph. The electrospray voltage was 2.1 kV. The intact peptides were detected in the Orbitrap at a resolution of 70,000, and the m/z full scan range was from 350 to 1800. Peptides were selected for MS/MS using an NCE setting of 28, and the fragments were detected in the Orbitrap at a resolution of 17,500. A data-dependent (DDA) was used to collect data, and the mother ions of the first 20 peptide segments with the highest signal intensity were selected to enter the HCD collision cell in turn for fragmentation with 28% fragmentation energy after the first-stage scanning and second-stage mass spectrometry. The 5E4 was set for automatic gain control (AGC), and the signal threshold and maximum injection time were set to 20,000 ions/s and 100 ms, respectively. The fixed first mass was set as 100 m/z [59, 60].

#### Bioinformatics analysis

The proteins were considered differentially expressed when the protein had both a log2-fold change of more than 1.50 or less than 0.67 and a *p*-value of less than 0.05 between intercropping and monoculture. Then, the gene ontology (GO) was created by searching the UniProt-GOA database (http://www.ebi.ac.uk/GOA/) [61]. Proteins were classified by GO annotation, and a two-tailed Fisher’s exact test was employed to test the enrichment of the differentially expressed protein against all identified proteins; the result was considered significant when the corrected *p*-value was less than 0.05 [61]. The Kyoto Encyclopedia of Genes and Genomes (KEGG) online service tool KAAS was used to annotate the proteins with KEGG database descriptions and to map the annotation results on the KEGG pathway database. A KEGG pathway with a *p*-value < 0.05 was considered significantly enriched [61].

#### PRM analysis

As described for the TMT analysis, the proteins of the root samples were extracted, reduced and digested with trypsin by using the EASY-nLC 1000 UPLC system. However, the electrospray voltage was 2.0 kV, and the full scan range was from 350 to 1100 m/z, which was different from the TMT analysis. In addition, the Orbitrap scanning resolution was set to 17,500. The 3E6 was set for AGC, and the maximum IT was set to 50 ms. The AGC of secondary mass spectrometry was set for 1E5, the maximum IT was set to 120 ms and the isolation window was set as 1.6 m/z [62, 63].

### Isotopic analyses: N transfer calculation and N derived from the transfer

All the materials (including roots, stem and leaves) from experiment 2 were dried at 65 °C and sifted using a 0.1 mm sieve to determine the ^15^N concentration using a mass spectrometer (SN09072D, Homotopic, Thermo Fisher Scientific, Germany). The value of plant ^15^N atom % excess was calculated using the following equation [64, 65].

The ^15^N content of receiver and donor leaf, stem and root were calculated as follows:

1 $$ {\mathrm{Excess}}^{15}\mathrm{N}{\mathrm{content}}_{\mathrm{compartment}}=\frac{\left(\mathrm{Aom}{\%}^{15}\mathrm{N} excess- Aom{\%}^{15}\mathrm{N}\ {excess}_{unlabelled}\ \right)\times \mathrm{Total}\ \mathrm{N}}{100} $$

The total excess ^15^N content of the whole plant was calculated by summing the excess ^15^N content in roots, stems and leaves.

The proportion of the total N in the receiver derived from the donor was calculated using the following equation

2 $$ \%\mathrm{NT}=\frac{{\mathrm{Excess}}^{15}\mathrm{N}\ {\mathrm{content}}_{\mathrm{receiver}}}{{\mathrm{Excess}}^{15}\mathrm{N}\ {\mathrm{content}}_{\mathrm{receiver}}+{\mathrm{Excess}}^{15}\mathrm{N}\ {\mathrm{content}}_{\mathrm{donor}}}\times 100 $$

where %NT is the percentage of total N transferred from donor to receiver.

The amount of N (mg plant^− 1^) transferred from the donor was calculated as follows:

3 $$ {\mathrm{N}}_{\mathrm{transfer}}=\frac{\%\mathrm{NT}\times \mathrm{total}\ {\mathrm{N}}_{\mathrm{donor}}}{100} $$

where N _transfer_ is the unidirectional net transfer from *D. odorifera* to *E. urophylla* × *E. grandis*.

### Statistical analysis

MS Excel and SPSS software were used for the data analyses. The statistical significance of differences between treatments was determined by analysis of variance (ANOVA) and least significant difference (LSD) multiple comparisons. The figures were created from the “gplots” R-package and SigmaPlot 13.0.

## Supplementary Information

**Additional file 1: Table S1.** Summary of the differential expressed proteins (> 1.5 times) in *E. urophylla × E. grandis* roots (*p* < 0.05) in this study.

**Additional file 2: Table S2.** Summary of the differential expressed proteins (> 1.5 times) in *D. odorifera* roots (*p* < 0.05) in this study.

**Additional file 3: Table S3.** KEGG annotation information of identified proteins of *E. urophylla × E. grandis* for the monoculture and intercropped treatments.

**Additional file 4: Table S4.** KEGG annotation information of identified proteins of *D. odorifera* for the monoculture and intercropped treatments.

## Data Availability

The data generated or analyzed in this study are included in this article and its supplementary information files. Other materials that support the findings of this study are available from the corresponding author on reasonable request.

## Abbreviations

EDTA: Ethylenediamine tetraacetic Acid
BCA: Bicinchoninic acid
RSD: Relative standard deviation
TMT: Tandem mass tag
SOD: Superoxide dismutase
PMR: Parallel reaction monitoring
LC-MS/MS: Liquid chromatography-tandem mass spectrometry
iTRAQ: Isobaric tags for relative and absolute quantitation
ADP: Adenosine-diphosphate
ATP: Adenosine-triphosphate
TCA: Tricarboxylic acid
AC: Acetyl-CoA
