# Supplementary material for: The physiological and molecular mechanisms of N transfer in Eucalyptus and Dalbergia odorifera intercropping systems using root proteomics
Source: BMC Plant Biol. 2021 Apr 26;21:201. doi: 10.1186/s12870-021-02969-9 (PMC8077921; doi:10.1186/s12870-021-02969-9)
Supplement: Supplementary file 3 — Additional file 3: Table S3. KEGG annotation information of identified proteins of E. urophylla × E. grandis for the monoculture and intercropped treatments. [file 12870_2021_2969_MOESM3_ESM.docx]

Table S3 KEGG annotation information of identified proteins of *E. urophylla × E. grandis* for the monoculture and intercropping treatments

| KEGG pathways | Number of protein | Related-protein accession |
| --- | --- | --- |
| Ribosome | 13 | A0A059D8G9, A0A059D2T8, A0A059CJI2, A0A059BXD8, A0A059CCR0, A0A059BJR3, A0A059B341, A0A059DHE6, A0A058ZVY2, A0A059DI03, A0A059DIW8, A0A059C7Z2, A0A059AVA7, |
| Phenylpropanoid biosynthesis | 9 | A0A059B094, A0A059DI26, A0A059CHH8, A0A059AJM6, A0A059AL91, A0A059A6N2, A0A059A000, A0A059C2H4, A0A059CG72 |
| Starch and sucrose metabolism | 6 | A0A059AEY0, A0A059BQS7, A0A059CH63, A0A059DI26, A0A059AN11, A0A059D4T9, A0A059A3G0 |
| Sesquiterpenoid and triterpenoid biosynthesis | 2 | A0A058ZSF8, A0A059CRD9 |
