# Supplementary material for: The physiological and molecular mechanisms of N transfer in Eucalyptus and Dalbergia odorifera intercropping systems using root proteomics
Source: BMC Plant Biol. 2021 Apr 26;21:201. doi: 10.1186/s12870-021-02969-9 (PMC8077921; doi:10.1186/s12870-021-02969-9)
Supplement: Supplementary file 4 — Additional file 4: Table S4. KEGG annotation information of identified proteins of D. odorifera for the monoculture and intercropped treatments. [file 12870_2021_2969_MOESM4_ESM.docx]

| KEGG pathways | Number of protein | Related-protein accession |
| --- | --- | --- |
| Spliceosome | 10 | TRINITY_DN47251_c0_g2\|m.32474, TRINITY_DN29346_c1_g1\|m.6645, TRINITY_DN33077_c0_g1\|m.9424, TRINITY_DN44104_c0_g1\|m.25170, TRINITY_DN43605_c0_g2\|m.24182, TRINITY_DN41960_c0_g1\|m.21108, TRINITY_DN44457_c0_g1\|m.25892, TRINITY_DN46632_c0_g1\|m.30822, TRINITY_DN43488_c1_g1\|m.23936, TRINITY_DN43935_c2_g1\|m.24797 |
| Flavonoid biosynthesis | 4 | TRINITY_DN25897_c0_g1\|m.4633 TRINITY_DN14556_c0_g1\|m.1020, TRINITY_DN60289_c0_g1\|m.42208 TRINITY_DN66001_c0_g1\|m.43347 |
| Glycosphingolipid biosynthesis-globo and isoglobo series | 2 | TRINITY_DN29960_c0_g1\|m.7119, TRINITY_DN47922_c0_g1\|m.34373 |
| Ubiquitin-mediated proteolysis | 4 | TRINITY_DN27771_c0_g1\|m.5701, TRINITY_DN38624_c0_g1\|m.15681, TRINITY_DN49484_c0_g1\|m.39497, TRINITY_DN42980_c0_g1\|m.22983 |
| Fatty acid degradation | 4 | TRINITY_DN46993_c0_g1\|m.31744, TRINITY_DN48560_c0_g1\|m.36376, TRINITY_DN48754_c1_g1\|m.36994, TRINITY_DN48102_c0_g2\|m.34960 |
| Protein processing in the endoplasmic reticulum | 8 | TRINITY_DN27771_c0_g1\|m.5701, TRINITY_DN29346_c1_g1\|m.6645, TRINITY_DN38624_c0_g1\|m.15681, TRINITY_DN44017_c0_g1\|m.24944, TRINITY_DN30307_c0_g1\|m.7328, TRINITY_DN29055_c0_g1\|m.6405, TRINITY_DN45138_c1_g2\|m.27418, TRINITY_DN49158_c0_g1\|m.38347 |

**Table S4** KEGG annotation information of identified proteins of *D. odorifera* for the monoculture and intercropping treatments
